# Translation of two-photon microscopy to the clinic: multimodal multiphoton CARS tomography of *in vivo* human skin

**DOI:** 10.1117/1.JBO.25.1.014515

**Published:** 2020-01-30

**Authors:** Karsten König, Hans Georg Breunig, Ana Batista, Andreas Schindele, Michael Zieger, Martin Kaatz

**Affiliations:** a JenLab GmbH, Berlin, Germany; b Saarland University, Department of Biophotonics and Laser Technology, Saarbrücken, Germany; c SRH Wald-Klinikum Gera GmbH, Gera, Germany

**Keywords:** multiphoton tomography, two-photon microscopy, coherent anti-Stokes Raman spectroscopy, femtosecond laser, medical imaging, second harmonic generation, fluorescence lifetime imaging, skin, psoriasis, dermatitis, nicotinamide adenine dinucleotide [NAD(P)H], translational medicine, label-free imaging, autofluorescence

## Abstract

Two-photon microscopes have been successfully translated into clinical imaging tools to obtain high-resolution optical biopsies for *in vivo* histology. We report on clinical multiphoton coherent anti-Stokes Raman spectroscopy (CARS) tomography based on two tunable ultrashort near-infrared laser beams for label-free *in vivo* multimodal skin imaging. The multiphoton biopsies were obtained with the compact tomograph “MPTflex-CARS” using a photonic crystal fiber, an optomechanical articulated arm, and a four-detector-360 deg measurement head. The multiphoton tomograph has been employed to patients in a hospital with diseased skin. The clinical study involved 16 subjects, 8 patients with atopic dermatitis, 4 patients with psoriasis vulgaris, and 4 volunteers served as control. Two-photon cellular autofluorescence lifetime, second harmonic generation (SHG) of collagen, and CARS of intratissue lipids/proteins have been detected with single-photon sensitivity, submicron spatial resolution, and picosecond temporal resolution. The most important signal was the autofluorescence from nicotinamide adenine dinucleotide [NAD(P)H]. The SHG signal from collagen was mainly used to detect the epidermal–dermal junction and to calculate the ratio elastin/collagen. The CARS/Raman signal provided add-on information. Based on this view on the disease-affected skin on a subcellular level, skin areas affected by dermatitis and by psoriasis could be clearly identified. Multimodal multiphoton tomographs may become important label-free clinical high-resolution imaging tools for *in vivo* skin histology to realize rapid early diagnosis as well as treatment control.

## 1 Introduction

Two-photon effects were predicted by the German PhD student Maria Göppert more than 90 years ago. The first paper was submitted on October 28, 1928, with the title “Über die Wahrscheinlichkeit des Zusammenwirkens zweier Lichtquanten in einem Elementarakt” (“On the probability of two light quantum working together in an elementary act”), and was published in 1929.^1^ Her PhD thesis, supervised by Max Born, was published in 1931.^2^ In 1930, she married the American Joseph Edward Mayer, who worked in Göttingen and moved to the United States. In 1960, Maria Goeppert-Mayer was appointed to a position as a full professor of physics at the University of California at San Diego (UCSD). It was the year the laser was invented. The novel intense light source provided the chance to prove her hypothesis of the simultaneous absorption by two photons, where the nonlinear absorption process scales with the square of the light intensity. In 1961, the first two-photon fluorescence was demonstrated by Kaiser and Garrett^3^ in laser exposed europium-doped crystals. In 1963, Maria Goeppert-Mayer became the second female Nobel laureate in physics. The unit GM for the characterization of two-photon absorption cross-sections ($1\;\mathrm{GM}=1\times 10^{50}\;{\mathrm{cm}}^{4}\;s\text{ photon}$) refers to her.

In 1988/1989, we realized in Jena the first ultrashort laser scanning microscope based on a tunable picosecond dye laser. The picosecond laser scanning microscope was used for fluorescence lifetime imaging (FLIM) in living cells and animals using time-correlated single-photon counting.^4^

Finally, 30 years ago in 1989/1990, the first two-photon microscope was developed by Denk et al.^5^ They demonstrated two-photon fluorescence in cells using a subpicosecond dye laser.

Using tunable near-infrared (NIR) femtosecond titanium:sapphire lasers, commercial two-photon laser scanning microscopes revolutionized modern live-cell 3D imaging microscopy at the end of the century. Typically, high numerical aperture (NA) objectives are employed to provide a “confocal-free” subfemtoliter two-photon excitation volume. With a typical *in situ* pulse width of 100 to 300 fs at the target, mean powers in the milliwatt range are used in 80-MHz titanium:sapphire laser microscopy of biological samples. The pulse width can be reduced by using pulse compression units (prism pairs, gratings, and chirped mirrors). When using sub-20 fs MHz laser pulses, microwatt mean powers are sufficient to perform two-photon imaging.^6^

It should be noted that two-photon effects such as second harmonic generation (SHG) and two-photon fluorescence can also be achieved with continuous-wave laser beams, however, the efficiency is extremely low compared to femtosecond laser pulses with transient kW peak powers.^7^^,^^8^

Femtosecond laser microscopes can also be employed to induce three-photon excited fluorescence,^9^ SHG,^10^ and third harmonic generation^11^ as well as nanoprocessing multiphoton tool.^12^

A major step was the translation of a two-photon microscope to a medical product (CE-certified 2a medical product). The first clinical multiphoton tomograph for high-resolution imaging in humans was built by König et al. in Jena, Germany, at the beginning of the new millenium.^13^^–^^15^

These novel multiphoton tomographs provide noninvasively marker-free optical biopsies under physiological conditions with a spatial resolution similar or better to conventional pathological examinations on extracted, sliced, and stained biopsies. Single cells and even intratissue intracellular organelles and single elastin/collagen fibers can be imaged with these novel femtosecond laser tomographs without any staining.

Multiphoton tomographs (DermaInspect, MPTflex, and MPTcompact) have been used in major clinics in Australia, Europe, and the United States (e.g., Princess Alexandra Hospital in Brisbane; Charité in Berlin; Policlinico of Modena, in Modena; Hammersmith Hospital in London; Hospital Saint-Louis in Paris; Waldklinikum Gera, in Gera; Eppendorf Clinic in Hamburg; and Beckman Laser Institute and Medical Clinic at University of California in Irvine) as well as in research centers of major cosmetic and pharmaceutical companies in Europe and Japan. Also, the skin of astronauts has been investigated after long-term space flights.^16^ So far, clinical multiphoton studies have been conducted on thousands of patients and volunteers (e.g., see Refs. 17–21).

A major development step was the introduction of a two-beam multiphoton tomograph for clinical coherent anti-Stokes Raman spectroscopy (CARS). The very first CARS study on humans was performed in 2010 with a CE certified clinical femtosecond tomograph by the dermatologists in the Charité, the largest hospital in the European Union, after approval by the ethics committee.^22^ The CARS tomograph was based on an optoacoustic modulator.

Further development included the modification into a compact flexible multimodal multiphoton/CARS tomograph that can be easily moved to the bed of a patient.^23^^–^^29^ For that purpose, the NIR beam of the tunable titanium:sapphire laser was tuned to 777 nm and split into two beamlets. One beamlet was transmitted through a photonic crystal fiber (PCF) for white-light generation with a spectral maximum of about 1-μm wavelength (Stokes beam). A challenge was the transmission of the two femtosecond laser beams (pump beam at 777 nm and “Stokes beam”) through the optical arm and their intratissue superposition in space and time.

Here, we present the results of the clinical study on *in vivo* multimodal multiphoton CARS tomography conducted in 2018 on 16 human subjects within a hospital using the compact two-beam multiphoton tomograph “MPTflex-CARS.” The tomograph was operated by medical staff members and not by laser engineers/laser physicists.

In particular, “atopic dermatitis”-affected and “psoriasis”-affected patients have been investigated. Atopic dermatitis (AD) is a type of inflammation of the skin with unknown cause, which affects up to 20% of people at some point in their lives.^30^ It causes the skin to become itchy, red, swollen, and cracked. Typically, AD is diagnosed based on observable signs and symptoms without special testing. The disease leads to a defective epidermal barrier but also deeper laying keratinocytes are affected in a complicated way.^31^^,^^32^ Psoriasis is an autoimmune disease leading to red, dry, itchy, and scaly skin. The most common form, the so-called “psoriasis vulgaris” (PV), manifests itself in red patches with a white scale.^33^ Diagnosis is typically based on the signs and symptoms, sometimes supplemented by skin biopsies. Characteristically, it leads to epidermal thickening and abnormal premature maturation of cells in the *stratum corneum*, where nuclei are present as a result of incomplete differentiation of corneocytes.^34^

As demonstrated in this clinical multiphoton imaging study, skin areas affected by dermatitis and psoriasis could be clearly identified.

## 2 Tomograph, Patients, and Methods

### 2.1 Multiphoton Tomograph

The tomograph MPTflex-CARS (JL DI MPT10 011 17) is based on an 80-MHz tunable titanium:sapphire laser (690 to 1040 nm) with an output power of >2  W. The laser wavelength was set to be 777 nm [excitation wavelength for nicotinamide adenine dinucleotide [NAD(P)H] autofluorescence and pump wavelength for CARS]. The spectral bandwidth is about 9 nm and $150\;{\mathrm{cm}}^{-1}$, respectively.

The beam was split into two beamlets with one beamlet transmitted through a special PCF for white-light generation (about 800 to 1100 nm).

A 950-nm longpass filter as beam combiner was chosen to use a broadband laser beamlet as Stokes beam to detect the well-known ${\mathrm{CH}}_{2}$-stretch vibrations (symmetric: $2845\;{\mathrm{cm}}^{-1}$ of lipids, antisymmetric: $2884\;{\mathrm{cm}}^{-1}$). This would require Stokes wavelengths of 997 and 998 nm, respectively. By passing a time delay unit and an articulated optical arm with active mirrors, the two beams overlapped in space and time within the focus of an NA1.3 objective.

The measurement that contains the beam scanning unit and the motor-driven focusing optics is also equipped with four photomultipliers with single-photon sensitivity for simultaneous detection of (i) autofluorescence intensity (405 to 565 nm) and lifetime (FLIM), (ii) SHG at 388.5 nm (BP390/40), (iii) CARS (636 nm, BP640/14), and (iv) “nonresonant background” (NR)-CARS (BP650/13, beamsplitter 650SP) to measure the NR in the neighboring spectral range around $2500\;{\mathrm{cm}}^{-1}$. It should be mentioned that certain crosstalk between the CARS signals from intratissue lipids and proteins in both channels (CARS and NR-CARS) cannot be avoided due to the poor resolution of femtosecond laser pulses (protein range, ${\mathrm{CH}}_{3}$ stretch: $2930\;{\mathrm{cm}}^{-1}$).

**Fig. 1:**
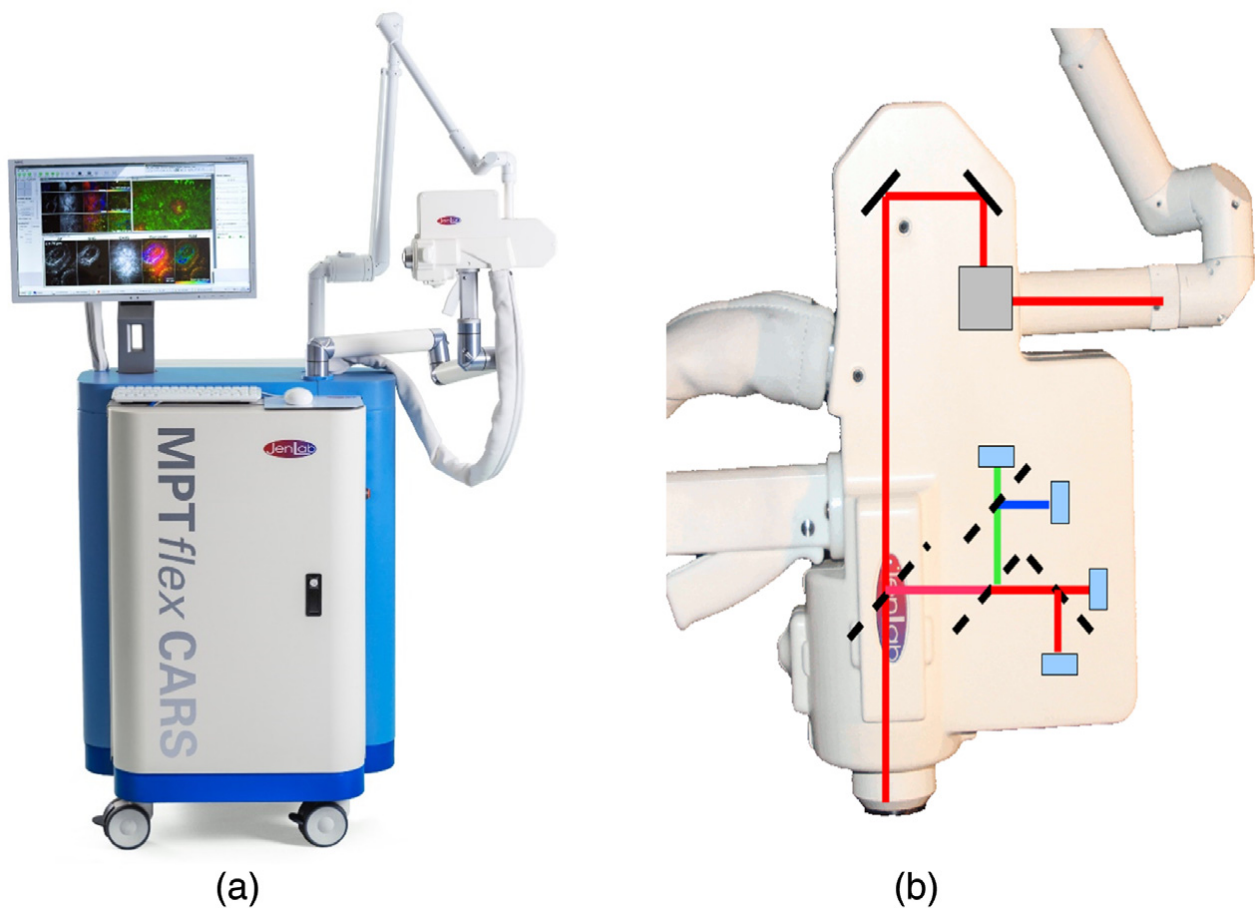
(a) Multiphoton tomograph MPT-CARS for imaging of two-photon AF, FLIM, collagen imaging by SHG, and lipid/protein imaging by CARS. (b) The 360 deg measurement head contains four detectors.

Figure 1 shows the two-beam tomograph MPT-CARS with its measurement head. The head contains four photomultiplier tubes (PMT); two are red-sensitive. The total mean power at the skin is limited to a maximum value of 50 mW due to the regulation by the certified body. Typically, mean laser powers between 2 and 20 mW are sufficient to detect endogenous intracellular fluorophores in the epidermis and backscattered SHG radiation from the upper dermis, respectively. The pump beam at 777 nm has sufficient power, however, the Stokes beam has, with respect to the spectral behavior of the specific PSF, a limited power at the desired wavelength at about 1  μm.

The power ratio of the pump beam to Stokes beam is 45  mW:5  mW=9:1. The piezo-driven and step-motor-driven focusing optics with an NA of 1.3 provides a maximum working distance of 200  μm.

Beam scanning is realized with x, y galvoscanners at typical beam dwell times of 2 to 16  μs/pixel. The field of view covers typically $360\times 360\;{\mu m}^{2}$ ($512\times 512\;{\text{pixels}}^{2}$). Optical zoom is possible. A resolution of <0.5  μm lateral and 1 to 2  μm axial can be realized for epidermal fluorescence. The interface between the high NA focusing optics (oil immersion) and the skin consists of a metal ring holding a special 160-μm-thick glass window.

**Fig. 2:**
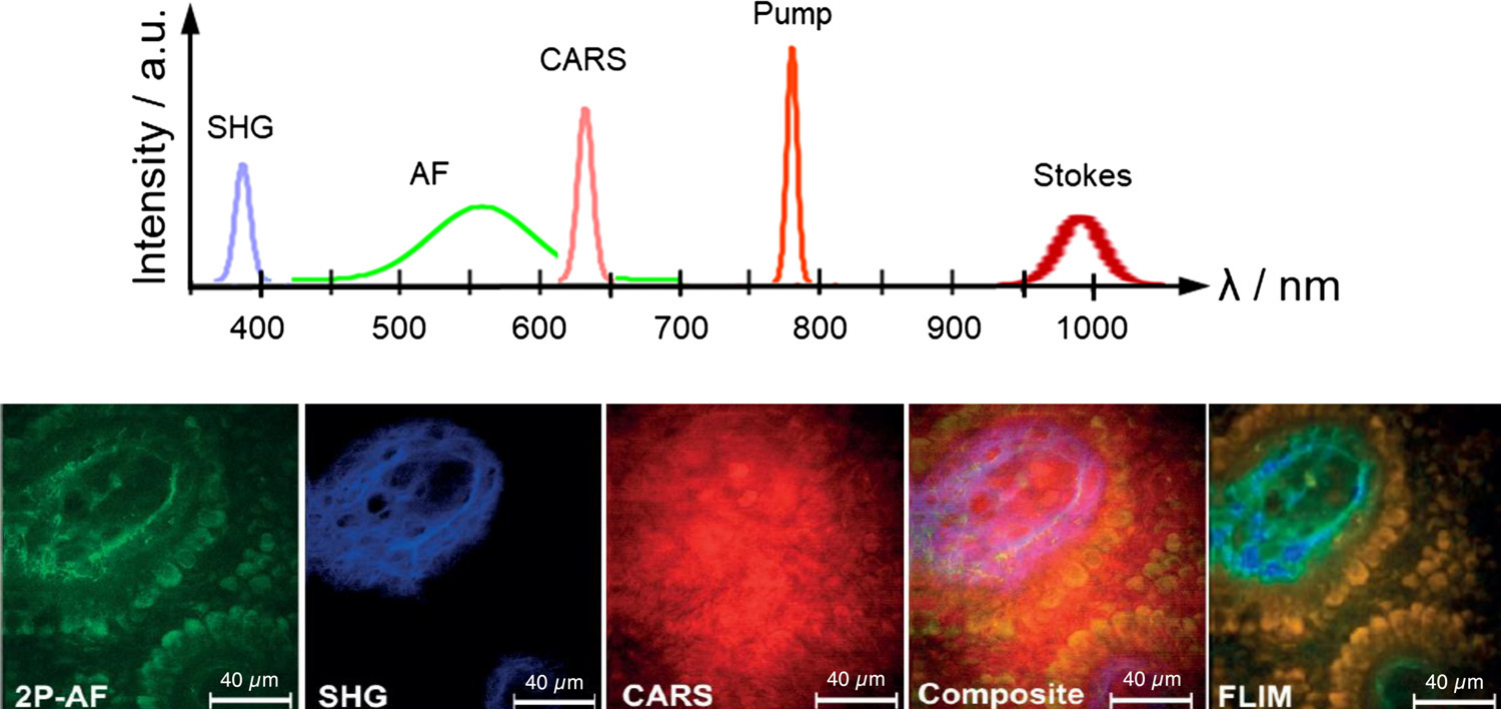
Scheme of multiphoton CARS tomography. Two ultrashort laser beams at 777 nm and a broadband Stokes at around 1  μm are employed to excite AF in the visible spectral range, SHG at half the laser wavelengths, and a CARS signal at 636 nm from the ${\mathrm{CH}}_{2}$-stretch vibration. One set up of multimodal images is depicted (modified from Ref. 33).

Figure 2 demonstrates schematic a spectrum with the three signals: autofluorescence (AF) [NAD(P)H maxima: 440 to 460 nm, flavins: 530 nm], SHG from pump beam at 388 nm and CARS at 636 nm as well as the wavelengths of the two ultrashort laser beams at 777 nm and 950–1050 nm. Furthermore, a typical en face section is shown with the three images of the three signals as well as the composite of the three images and a calculated pseudocolored FLIM image (modified from Ref. 27).

### 2.2 Patients

The clinical study titled “Prospective monocentric controlled study for the evaluation of the diagnostic potential of *in vivo* coherent anti-Stokes Raman spectroscopy for psoriasis vulgaris and atopic dermatitis (CARS-PAD)” was permitted by the authorities in Germany (EUDAMED No: CIV-17-12-022506).

Patients older than 18 years presenting with clinically suspected AD and PV at the Department of Dermatology, Waldklinikum Gera, in Gera, were included. The patients were recruited in conformity with the Declaration of Helsinki and informed about the procedures and the risks. They gave written informed consent. Initial clinical assessment, most times including dermoscopy, was performed by experienced dermatologists.

The clinical study included six patients affected by AD with ages comprised between 19 and 78, six patients suffering from psoriasis aged between 48 and 62, and four healthy volunteers who were between 27 and 53 years old.

### 2.3 Methods

Multiphoton tomography (MPT) on the 16 human subjects was performed completely label-free. In preparation for the measurements, the skin was cleaned. A cover glass inside a metal ring with a thin layer of water underneath and immersion oil on top was put onto the skin to reduce reflections at the interface. For each subject, three nonoverlapping volumes of the affected area or of the forearm (healthy skin) were imaged by *en face* optical sectioning in z-steps of 5  μm covering $160\times 160\times 100\;\mu m^{3}$. It took up to 30 min for the patient to be measured.

## 3 Results and Discussion

**Fig. 3:**
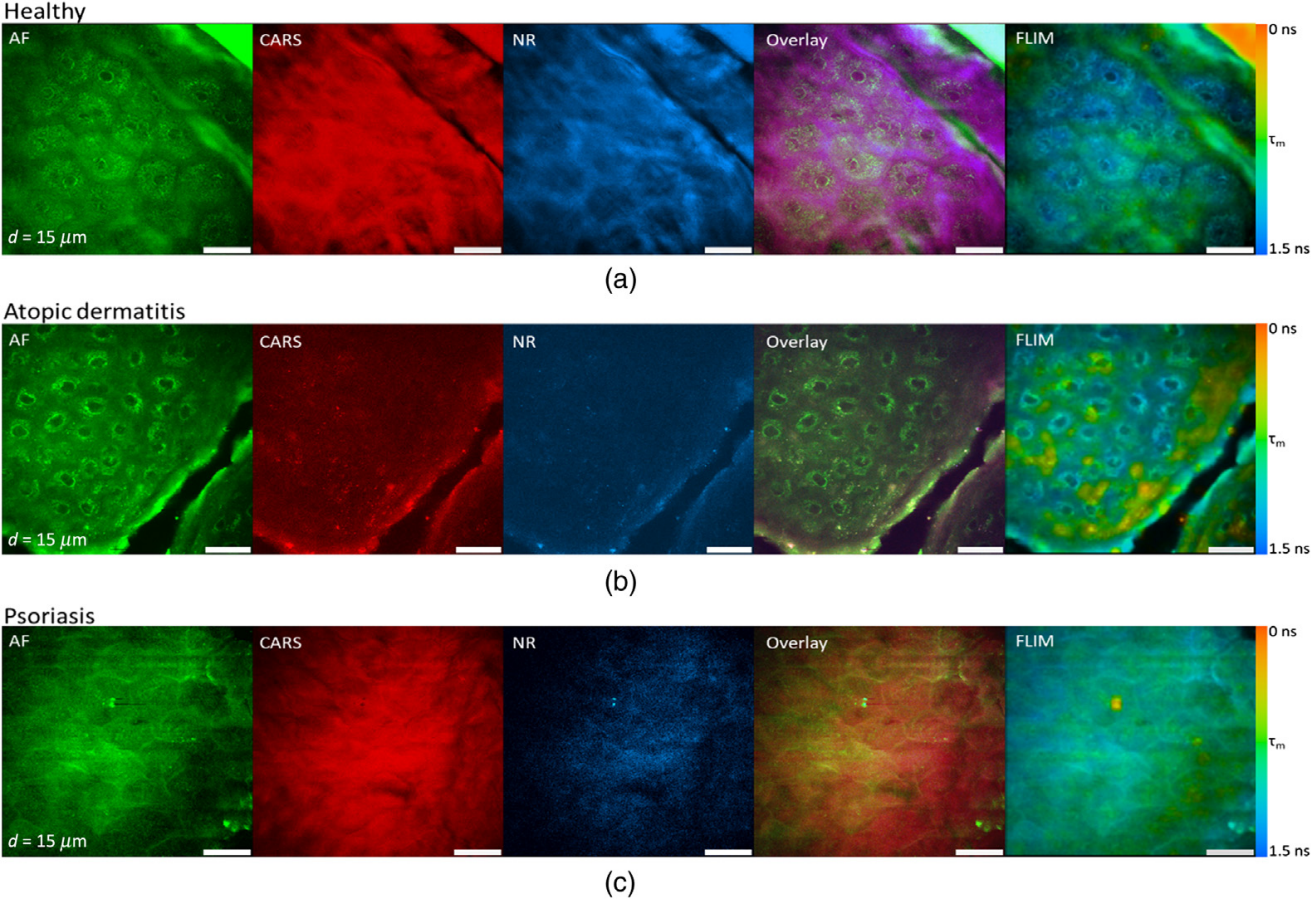
Multiphoton sections out of a 3D stack of an optical biopsy of the arm of (a) a healthy patient compared to the skin of patients suffering from (b) AD and (c) psoriasis.

In order to illustrate lesions of healthy- and disease-affected skin, multimodal nonlinear *in vivo* imaging combining AF intensity, AF lifetime, SHG, NR, and CARS has been applied. Typical results from a depth d=15  μm are shown in Fig. 3. The figure shows overlay images to visualize the signal contributions of different modalities in false colors. SHG signals are not present due to the lack of SHG-generating structures inside this skin layer, the *stratum spinosum*. The FLIM images generally reaffirm the morphology of the fluorescence intensity images and indicate, in addition, the fluorescence lifetimes, which are characteristics for the involved molecules.

### 3.1 Healthy Skin

From the endogenous skin fluorescence (Fig. 3a; AF), keratinocytes are clearly distinguishable by their round shapes and their dark-appearing nuclei, which do not contain fluorescent material. The cell autofluorescence mainly stems from NAD(P)H. The CARS and NR images clearly indicate structural morphology but with less spatial resolution and contrast than the AF image taken with the short 777-nm wavelength. In the overlay image (Fig. 3a; overlay) autofluorescence, CARS and NR signals are combined. The CARS and NR signal distributions highlight roughly the cell membranes. CARS signals, in general, contain resonant and background contributions. Different strategies have been developed to distinguish both contributions and correct for the NR.^35^^,^^36^

As a simple but straightforward approximation of the NR signal strength, we recorded the NR signal inside a neighboring spectral window with the same spectral width as the one used to record the lipid signals at 636 nm, as shown in the NR column in Fig. 3. This method does not account for the interference between NR and resonant-signal contributions, which are contained in the CARS signal. Nevertheless, it makes a rough comparison of resonant and NR signal strengths possible. By comparison of the corresponding images, i.e., the CARS images (lipid/protein signal plus NR plus interference term) and the NR images, the assumption can be drawn that the actual lipid contribution is small and that the NR is a suitable contrast mechanism to indicate cell boundaries or typical hexagonal shapes in the stratum corneum (Fig. 3c; CARS).

We can exclude the contribution of red autofluorescence, such as from porphyrins produced by the skin bacterium *Propionibacterium acnes*, due to control measurements where the time delay between the two NIR femtosecond laser beams was changed. CARS signals appear only when both beams are superimposed in space and time, whereas the fluorescence does not require both beams simultaneously.

The FLIM image of the healthy viable epidermis indicates mean decay times of 1.5 ns or longer inside the cells, i.e., typical values for a mixture of free and bound NAD(P)H molecules.^15^

### 3.2 Disease-Affected Skin

In lesions affected by AD, strong autofluorescence signals were detected. The cell morphology here differs from that of healthy cells, which clearly indicate pathology-induced changes resulting from intercellular edema and perinuclear accumulation of the NAD(P)H-containing mitochondria, i.e., apparently stronger signals close to the nuclei. These changes have been observed previously and thoroughly evaluated.^22^ The CARS and NR signals exhibit as in the case of healthy skin similar intensities. Therefore, the overlay image is clearly dominated by the autofluorescence signals.

In the psoriasis-affected lesion, the thickening of the epidermal layer, i.e., in particular, the enlarged stratum corneum prevented reaching the cellular layers at the recorded depth; no cells are visible (Fig. 3c). The psoriasis-affected skin also exhibits strong CARS signals, somehow weaker NR signals (Fig. 3c; NR), which reflect the hexagonal shape of cells in the stratum corneum.

**Fig. 4:**
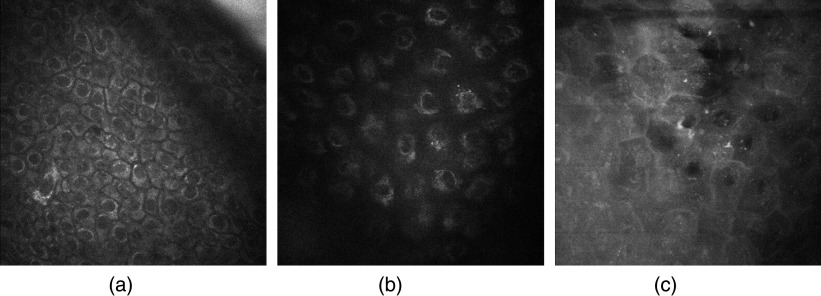
Typical autofluorescence images of the epidermis at a depth of 30  μm. (a) Healthy patient, (b) AD patient, and (c) PV patient.

**Fig. 5:**
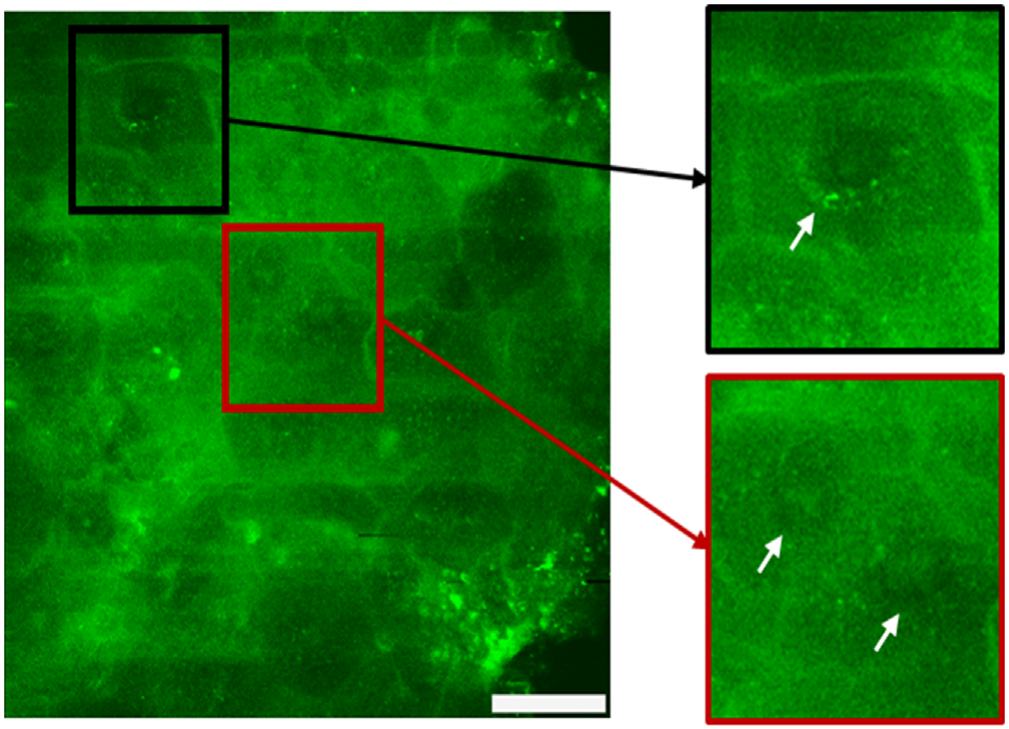
AF image of the stratum corneum of a patient suffering from psoriasis. Note the presence of cells with nucleus in the outermost layer stratum corneum.

Figure 4 shows typical AF patterns. The cell–cell distance and the distribution of the mitochondria within the cytoplasm differ in dependence on the deceased. In particular, in the case of AD, mitochondria are located as “ring structure” close to nucleus border as a typical sign of inflammation. Figure 5 highlights the AF pattern of psoriasis patients. In contrast to healthy skin, here, i.e., inside the thickened stratum corneum, even cell nuclei can be distinguished as a result of the pathologically accelerated cell lifecycles. These are discernable with even more clarity even close to the skin surface. The figure shows an autofluorescence intensity image recorded at a depth of 10  μm inside the stratum corneum. The presence of nuclei is a consequence of the psoriasis-induced abnormal premature maturation of the keratocytes leaves the cells not enough time to clearly differentiate into anucleated corneocytes.

**Fig. 6:**
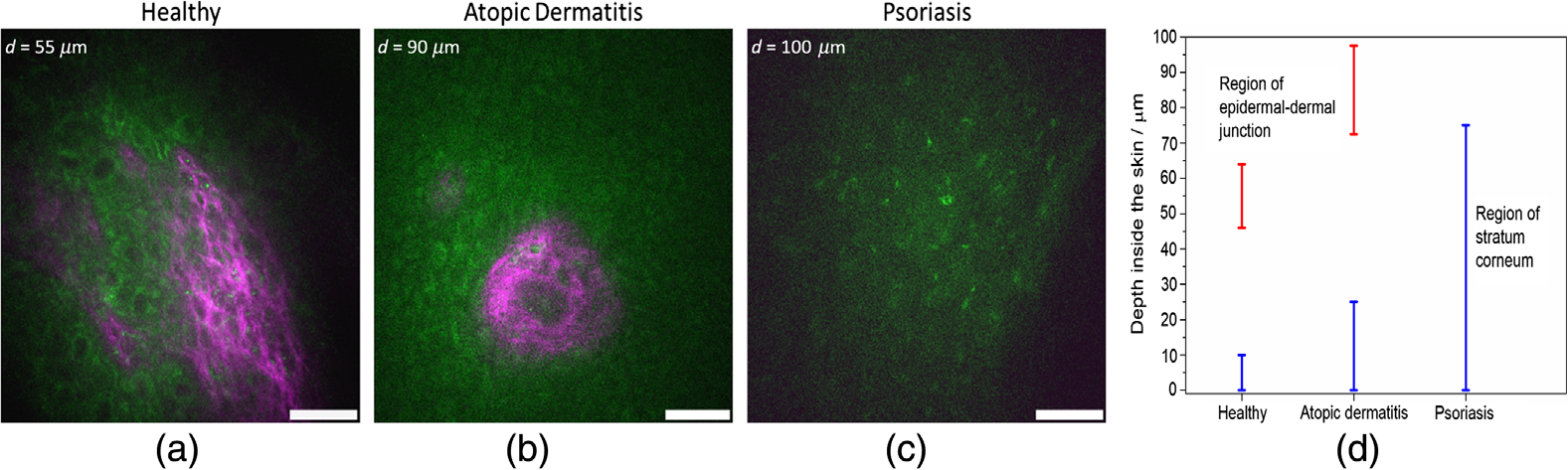
Thickness of the stratum corneum and the depth of the EDJ: (a)–(c) SHG images and (d) calculated mean values. The EDJ is deeper in patients suffering from AD and psoriasis. The first signals of SHG occur in a depth of about 50  μm in healthy skin but in depths larger than 50  μm in the cases of AD and PV.

Typical images from depths where healthy skin forms the epidermal–dermal junction (EDJ) are shown in Fig. 6. SHG contributions stemming from the collagen network become visible at a depth of about 50  μm, which is typical for healthy skin at the forearm. Also, in the skin affected by AD, a typical papilla consisting of collagen (and non-SHG generating fluorescent elastin) is visible. Nevertheless, due to the epidermis thickening caused by this pathology, the EDJ is only reached in this case at a depth of about 90  μm.

In Fig. 6(c), the thickening of the epidermis makes the epidermal–dermal region unreachable within the maximum depth of the stacks. Even at a depth of ∼100  μm, the image indicates a “stratum corneum-like” features without SHG contributions. To illustrate that further, the depth ranges of the stratum corneum and the depths of the EDJs for all lesions, healthy- and disease-affected, are summarized in the scheme. The figure clearly indicates the pathology-induced thickening of the epidermis for both pathologies, which is, in particular, pronounced in psoriasis-affected patients.

**Fig. 7:**
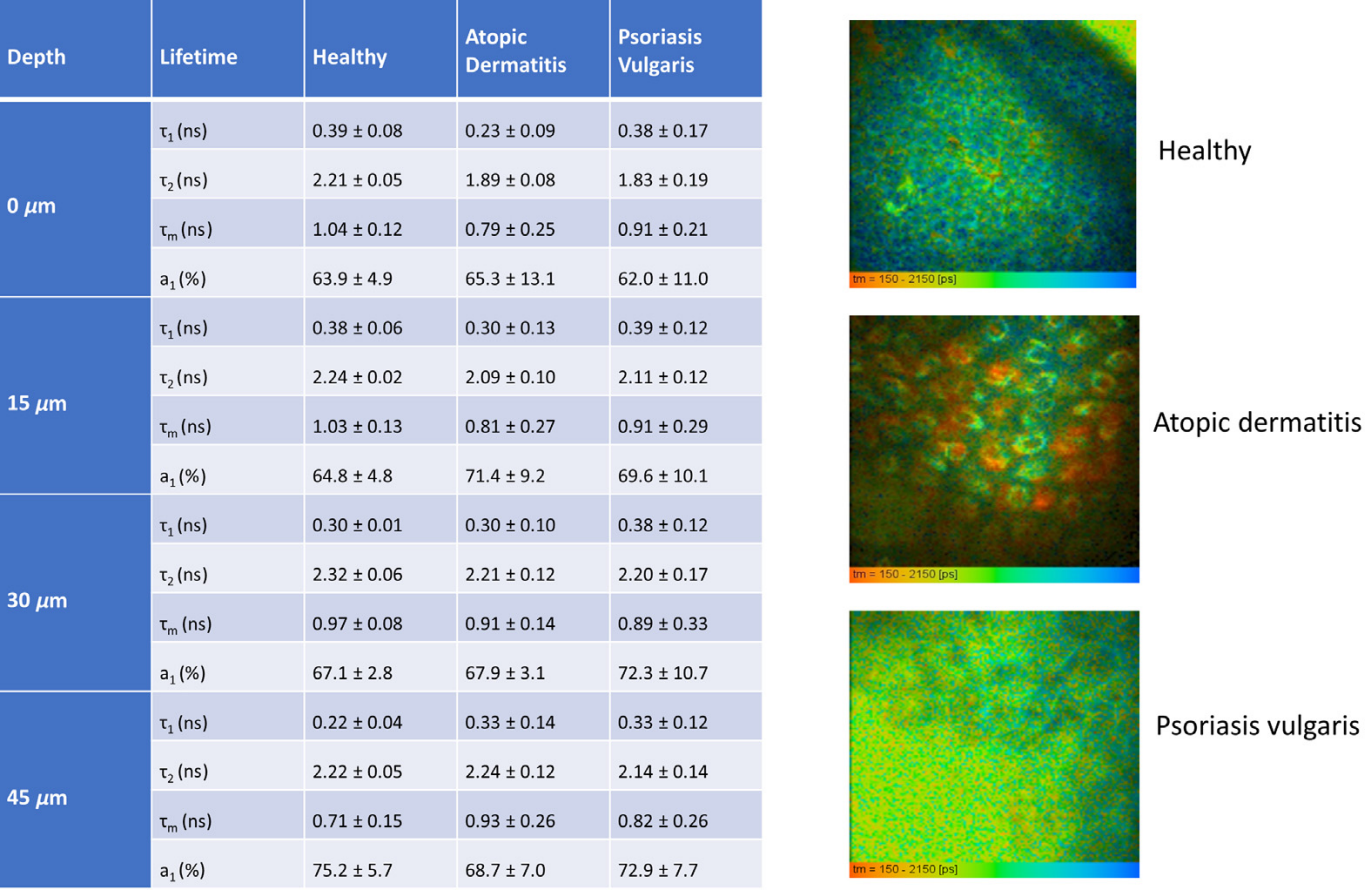
Multiphoton fluorescence lifetime imaging. The mean fluorescence lifetime per pixel can be depicted as pseudocolored FLIM image. The figure represents a typical optical FLIM section in 40-μm skin depth for a healthy volunteer compared with AD and PV.

**Fig. 8:**
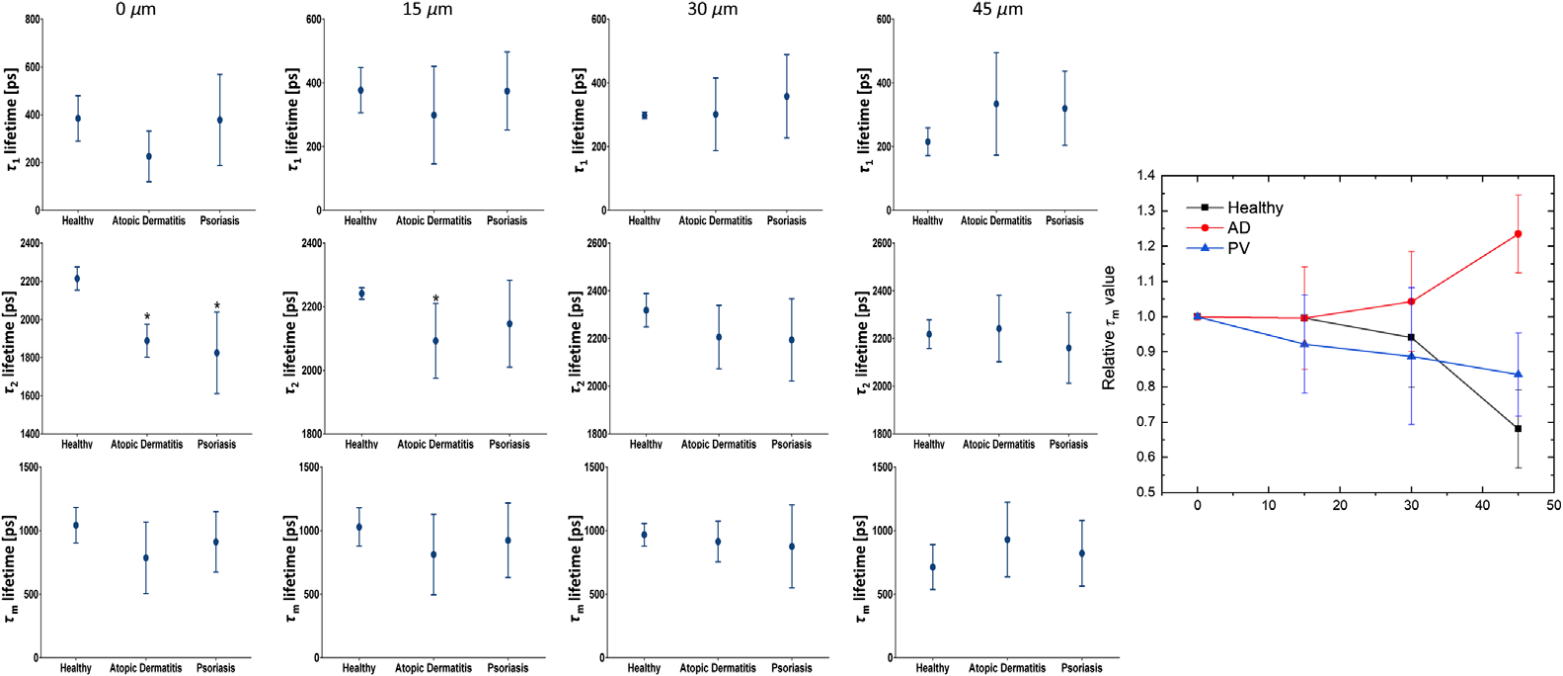
Average fluorescence lifetimes ($\tau_{1}$, $\tau_{2}$, and $\tau_{m}$) and variations in dependence on skin depth and deceased. The scheme shows the significant modifications of the mean fluorescence lifetimes when going deep depicted as relative $\tau_{m}$ values. The values increase in the case of AD in contrast to healthy tissue. The mean lifetime of PV is determined by keratin in the enlarged stratum corneum.

The mean fluorescence lifetime is about 1 ns in the first 30-μm skin depth in the healthy skin and decreases when going deeper (Figs. 7 and 8). This decrease can be explained with the presence of melanin mainly in the “stratum basale” with its short fluorescence lifetime.

In the case of psoriasis, the fluorophore keratin is present in the first 50  μm and not influenced by melanin due to the enhanced thickness of the stratum corneum.

Interestingly, the fluorescence lifetimes vary most in the case of skin affected by AD. The lifetime even increases when going deeper (Fig. 8). However, within the first 30-μm skin depth, the mean lifetimes are lower than in healthy skin, a trend in line with findings by Huck et al.^22^

**Fig. 9:**
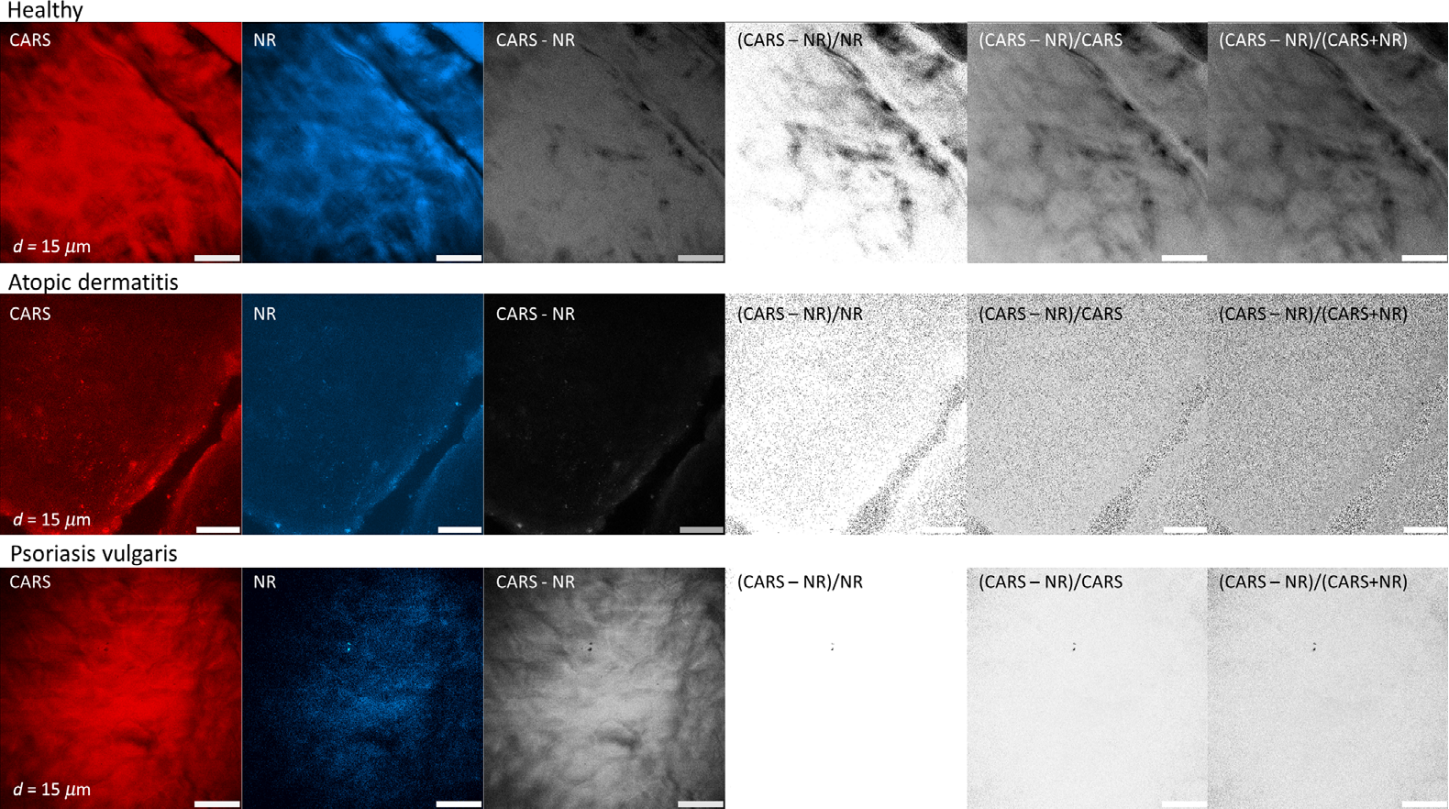
Clinical CARS images of healthy subjects compared to patients suffering from AD and psoriasis. Also, the nonresonant background (NR) is depicted and the CARS images obtained after NR subtraction and ratio calculations.

Figure 9 highlights the CARS signal and the “nonresonant background” (NR). Both demonstrate similar features. As already mentioned, the NR signal may still contain CARS signals from skin origin. We hoped to gain more information when subtracting the NR from the signal, however, a typical “lipid pattern” in dependence on the type of skin disorder was not found. The signal contrast can be enhanced when dividing the (CARS-NR) value by the CARS value or the (CARS + NR) value. However, no significant deceased-specific pattern was found.

It should be mentioned that the lipid concentration in keratinocytes is of magnitudes smaller than in adipocytes. Therefore, it seems difficult to image skin lipids with femtosecond Stokes beams and femtosecond pump laser beams (femto–femto CARS).

Nevertheless, the CARS modality was found earlier to be useful to detect lipids in fat cells and to detect exogenous lipids in topically applied pharmaceuticals and cosmetics.^23^^–^^30^

## 4 Conclusion

The two-beam multiphoton/CARS tomograph has been successfully tested in a clinical trial involving patients suffering from AD and PV. The results indicate that label-free multimodal multiphoton imaging is a suitable noninvasive method to directly obtain clinic *in-vivo* microscopic information on deceased skin with sufficient spatial resolution (optical biopsy). The multimodal imaging, in particular, of two-photon autofluorescence and SHG of collagen, enables visualization of complementary features like the cell–cell distance, the ratio nucleus/cytoplasm, the distribution of intratissue mitochondria, cell boundaries, and the depths of the EDJ. In fact, typical features of skin affected by AD and PV can be seen in “real time” on the screen during optical sectioning of the epidermis.

The measurement of the fluorescence lifetime in particular from the reduced coenzyme provides metabolic information. Significant differences in FLIM data have been monitored between the three groups (AD, PV, and healthy) that have the potential for an early diagnosis of skin diseases.

The CARS images taken with the moveable multimodal multiphoton/CARS tomograph by medical personal in a clinical environment (hospital) from patients with dermatological disorders did not meet our expectations based on previous CARS studies on CARS probes, biopsies, murine tissues, and volunteers. When comparing the different multiphoton signals within this clinical study, the autofluorescence images provided by far the best contrast and the best spatial resolution.

Two-photon autofluorescence imaging and SHG imaging can be performed best with femtosecond laser pulses rather than with picosecond pulses. However, CARS imaging can be better performed with picosecond laser pulses due to higher spectral resolution that is, in particular, helpful when detecting certain chemicals with Raman signals in the fingerprint region. Our state-of-the-art clinical “femto/femto CARS system” is probing ${\mathrm{CH}}_{2}$ and ${\mathrm{CH}}_{3}$ stretches due to the broad spectral band (FWHM vibrational coverage about $150\;{\mathrm{cm}}^{-1}$). Therefore, also the “CARS-NR” channel contains significant biological information from lipids/proteins and the subtraction method did not provide a benefit. Interestingly, the background signal is also helpful to localize tissue structures. This was also monitored by Saar et al.^36^ when comparing background-free stimulated Raman scattering (SRS) with CARS on murine tissue. The SRS image of intratissue water provided a homogenous signal without any gray level fluctuations (no contrast), whereas the CARS water image depicted also the cell membranes. It should be noted that so far, no clinical SRS system exists. SRS would require additional hardware including high-frequency modulation lock-in technique, and large field sensors that may face problems due to electromagnetic noise regulations and other medical product requirements. First SRS images of live human skin treated with deuterium-labeled dimethyl sulfoxide with a unique vibration at $2120\;{\mathrm{cm}}^{-1}$ that is normally used as solvent in NMR spectroscopy have been obtained from the forearm of a volunteer using a home-built all-analog lock-in amplifier with 100-ns response time in combination with a 20-MHz modulated Stokes beam and a large-area epidetector in front of the objective of the lab microscope.^36^

A good compromise for clinical nonlinear two-beam multimodal imaging (AF, SHG, and CARS) with a focus on high spatial resolution of intratissue cells is the use of NIR ultrashort laser radiation, where at least one laser beam should have a pulse width in the femtosecond range and a short NIR wavelength of 800 nm or less to image the most important endogenous fluorophore NAD(P)H with high spatial resolution.

Besides medical diagnostics, femtosecond or picosecond CARS/SRS on humans^36^^–^^38^ is of interest for the measurement of the intradermal water content, to trace sebaceous glands, and to gain information on the intratissue distribution of topically applied lipid-rich cosmetics and pharmaceutics, including probing the stratum corneum barrier function.

Further investigations with a higher number of patients are needed to confirm the observed differences in autofluorescence, SHG, and CARS within this clinical study on 16 subjects and distinguish possibly more subtle features of individual courses of the skin diseases. For that case, we started to employ artificial intelligence/deep learning procedures.^39^^,^^40^

We hope that multimodal MPT will provide a new insight into pathological effects on a molecular and subcellular scale *in-vivo* and may lead to a future noninvasive, rapid, and label-free diagnostic procedure.
